# Factors Affecting Wound Healing in Patients with Venous Leg Ulcers: A Pilot Study

**DOI:** 10.3390/nu18071148

**Published:** 2026-04-03

**Authors:** Hubert Aleksandrowicz, Joanna Czerwińska, Waldemar Placek, Agnieszka Owczarczyk-Saczonek

**Affiliations:** 1 Dermedica, Aleja Zwycięstwa 34, 80-219 Gdańsk, Poland; 2 Department and Clinic of Dermatology, Sexually Transmitted Diseases and Clinical Immunology, University of Warmia and Mazury, Al. Wojska Polskiego 30, 10-959 Olsztyn, Poland; joanna.czerwinska@uwm.edu.pl (J.C.); w.placek@wp.pl (W.P.); agnieszka.owczarczyk@uwm.edu.pl (A.O.-S.)

**Keywords:** malnutrition, micronutrients, vitamins, venous leg ulcers, wound healing, clinical trials

## Abstract

**Background/Objectives:** Previous studies have compared nutritional deficiency parameters in patients with venous leg ulcers (VLUs) to healthy individuals or those with unrelated conditions. This single-center study assessed blood levels of factors involved in ulcer healing and compared patients with VLUs to those with chronic venous insufficiency without ulcers. **Methods**: A total of 24 patients were included: 17 with VLUs formed the study group, and 7 with lower-limb venous insufficiency without ulcers served as controls. Disease severity was assessed using the CEAP classification, and all participants underwent ankle–brachial index (ABI) measurement. Venous blood samples were analyzed for selected vitamins, proteins, ions, protein electrophoresis, and amino acid profiles. **Results:** Strong correlations were identified (r > 0.5 or r < −0.5), some of which were statistically significant. Positive associations in the study group included BMI with waist circumference (r = 0.85, *p* < 0.05), tyrosine with proline (r = 0.84, *p* < 0.05), and valine with leucine (r = 0.82, *p* < 0.05). Negative correlations included albumin with folic acid (r = −0.73, *p* < 0.05), albumin with vitamin B6 (r = −0.71, *p* < 0.05), and folic acid with waist circumference (r = −0.65, *p* < 0.05). No significant differences in blood concentrations were observed between groups. **Conclusions**: Statistically significant correlations were observed between selected biochemical parameters, including albumin and alpha-1 globulins, as well as amino acid and vitamin concentrations, in both patients with VLUs and controls with chronic venous insufficiency without ulcers. Larger studies are needed to confirm these findings and clarify their relevance to venous leg ulcers.

## 1. Introduction

Existing studies have investigated concentrations of selected wound-healing factors in patients with venous leg ulcers (VLUs). These factors have been compared among patients with VLUs, healthy individuals, and patients with other diseases. However, comprehensive analyses of these factors and their interrelationships across different stages of chronic venous insufficiency are lacking [1,2,3,4,5,6,7,8,9,10,11,12,13,14,15,16].

Chronic venous insufficiency of the lower limbs affects approximately 60% of adults and leads to leg ulcers in 0.3–3% of cases [17,18,19,20,21]. Healing of venous leg ulcers typically requires 6–12 months and is associated with a high recurrence rate [21,22,23], with approximately 70% of ulcers recurring within five years [18,22,23]. Healing time may also depend on the patient’s nutritional status.

Effective management requires standard treatment combined with strategies addressing disease-related risk factors and optimizing nutritional status [1,2,3,4,5,6,7,8,9,10,11,12,13,14,24,25,26,27,28,29,30,31,32,33,34,35,36,37,38,39,40,41,42,43,44]. Intensive management, including a high-protein and energy-adequate diet, may improve treatment outcomes. However, nutritional aspects are often overlooked in clinical practice, which can negatively affect healing [24,27,31,38].

Water-soluble vitamins and minerals act as cofactors in metabolic processes involved in wound healing. Adequate intake of proteins, amino acids (such as arginine and glutamine), vitamins A, B-complex, C, D, and E, and minerals including zinc, copper, and iron is essential for tissue repair (Table 1). Deficiencies in these nutrients may delay the healing of chronic wounds [25,26,27,28,29,30,31,32,33,34,35,36,37,38,39,40,41,42,43,44]. Zinc and vitamin C are particularly recommended due to their roles in collagen synthesis and regulation of inflammatory processes [41,42].

This study was conducted as an observational exploratory investigation. Its primary objective was to preliminarily assess potential differences in selected biological parameters between patients with VLUs and a control group of patients with venous insufficiency without leg ulcers, and to explore associations between the investigated markers and selected clinical parameters. The study was designed as an observational investigation with exploratory analyses. A single blood sample was collected from each participant, and biological parameters were assessed at a single time point. The primary objective was to evaluate differences in selected biological parameters between patients with venous leg ulcers and a control group with chronic venous insufficiency without ulcers, as well as to explore potential associations between the investigated markers and selected clinical parameters in both groups. The study was approved by the Bioethics Committee, and written informed consent was obtained from all participants prior to enrollment.

## 2. Materials and Methods

### 2.1. Study Population

This single-center study, conducted between October 2021 and October 2023, included patients with venous insufficiency ranging from C1 to C6 according to the Clinical–Etiology–Anatomy–Pathophysiology (CEAP) classification. The CEAP classification is a standardized system used to categorize chronic venous insufficiency of the lower limbs. It includes four components: C (clinical presentation), E (etiology), A (anatomical distribution), and P (pathophysiological mechanisms). The clinical component (C) is organized according to increasing disease severity, with classes ranging from C0 to C6. Higher classes indicate more advanced stages of chronic venous disease and may include features observed in lower classes. Class C0 indicates no visible or palpable signs of venous disease. Class C1 includes telangiectasias and/or reticular veins. Class C2 denotes varicose veins. Class C3 is characterized by edema. Class C4 includes skin changes related to venous disease, such as pigmentation, venous eczema, or lipodermatosclerosis. Class C5 indicates a healed venous ulcer, while class C6 denotes an active venous ulcer [46].

Participants were recruited from the Outpatient Clinic and the Dermatology Department at the University of Warmia and Mazury in Olsztyn. Participation in the study was offered to patients identified from the clinic’s database, who were contacted and invited to take part. Individuals who expressed willingness to participate and met the inclusion criteria were subsequently allocated to the appropriate study groups.

A total of 24 patients were enrolled: 17 with venous leg ulcers (study group) and 7 with venous insufficiency without ulcers (control group). The control group included three patients at CEAP C5, two at C3, one at C1, and one at C2.

Moreover, demographic characteristics, including age and sex, as well as anthropometric parameters such as body mass index and waist circumference, were collected. Each participant underwent assessment of the ankle–brachial index and venous blood sampling to determine concentrations of vitamins, proteins, and ions, as well as to perform proteinogram and amino acid profile analyses. Information on comorbidities that may affect the healing of venous ulcers, such as diabetes, renal insufficiency, and hypothyroidism, was obtained based on medical history.

### 2.2. Study Design

The inclusion criteria included patients aged 18 years or older with venous leg ulcers (VLUs) and an ankle–brachial index (ABI) between 0.90 and 1.30. Patients were excluded if they had an ABI below 0.90 or above 1.30, a history of malignancy, active infections, or were pregnant or lactating. The study group comprised patients with venous leg ulcers, whereas the control group included patients with lower limb venous insufficiency without ulceration. The severity of venous insufficiency was assessed clinically using the CEAP classification. All clinical data were collected by the same investigator based on direct patient examination. Serum concentrations of factors influencing wound healing were measured, and blood samples for laboratory analyses were collected according to a standardized protocol. All laboratory tests were performed in the same certified laboratory using routine diagnostic methods. Patient confidentiality was strictly maintained, and personal data were not included on laboratory request forms. Standard pre-analytical conditions were ensured, including blood sample collection in the morning (08:00–10:00) from fasting participants, with all samples obtained in the same room. Anonymized laboratory results were subsequently provided to a single investigator responsible for the assessment of the study participants.

### 2.3. Clinical Assessment

Clinical assessment of lower limb venous insufficiency was performed through a thorough physical examination. Particular attention was given to the presence or absence of telangiectasia and visible or palpable varicose veins. Additional features evaluated included ankle edema, active or healed venous ulcers, and skin and subcutaneous changes secondary to venous insufficiency, such as pigmentation, eczema, lipodermatosclerosis, atrophie blanche, and corona phlebectatica. Patients were classified according to the CEAP system based on these findings. The clinical assessment was conducted before measuring the ankle–brachial index (ABI).

### 2.4. Ankle–Brachial Index

Each patient underwent measurement of the ankle–brachial index (ABI). Before blood pressure assessment, patients rested in the supine position for 10 min and remained lying down throughout the procedure. Blood pressure cuffs were applied to the arms at heart level and just above the ankles. Ultrasound gel was placed over the brachial pulse in the antecubital fossa and over the dorsalis pedis and posterior tibial arteries. Systolic pressures were measured using a handheld 8-MHz Doppler probe, Hadeco, Kawasaki, Japan. After identifying the arterial Doppler signal by placing the transducer over the gel, the cuffs were inflated until the signals were no longer audible and then slowly deflated until the signals reappeared. Measurements began with the right brachial artery, followed by the right dorsalis pedis and posterior tibial arteries. Systolic pressures were then recorded from the dorsalis pedis and posterior tibial arteries of the left leg, followed by the left brachial artery. All measurements were documented, and ABI values were calculated and reported to two decimal places.

### 2.5. Blood Samples

Serum concentrations of factors relevant to wound healing were measured, including vitamins—vitamin A (retinol), vitamin B1 (thiamine), vitamin B2 (riboflavin), vitamin B6 (pyridoxine), vitamin B9 (folic acid), vitamin B12 (cyanocobalamin), vitamin C (ascorbic acid), 25-hydroxyvitamin D3 [25(OH)D3], and vitamin E (tocopherol)—and proteins, including C-reactive protein (CRP), hemoglobin (Hb), glycated hemoglobin (HbA1c), ferritin, thyroxine (FT4), and triiodothyronine (FT3). A proteinogram and amino acid profile were also performed. Blood analyses further included measurements of ions (zinc, copper, and iron) and other relevant substances, such as beta-carotene, homocysteine, creatinine, and uric acid.

### 2.6. Statistical Analysis

All analyses were performed using statistical software (STATISTICA, version 13; StatSoft Inc., Tulsa, OK, USA). Results are presented as mean ± standard error of the mean (SEM). The distribution of continuous variables was assessed for normality using the Shapiro–Wilk test, and homogeneity of variance was evaluated with Levene’s test. For sociodemographic variables (age and BMI), the assumptions of normality and homogeneity of variance were met; therefore, comparisons between groups were performed using Student’s *t*-test for independent samples. For all other variables, the non-parametric Mann–Whitney U test was used. Correlation analyses were conducted according to data distribution, with Spearman’s rank correlation coefficient applied for non-parametric variables. Differences were considered statistically significant at *p* < 0.05.

## 3. Results

Demographic data, including age and sex, were collected, comprising 13 males and 11 females. The mean age of participants was 64.75 ± 2.62 years. Anthropometric measurements, including height, body weight, and waist circumference, were recorded. The mean waist circumference was 110.36 ± 5.09 cm for females and 137.15 ± 10.8 cm for males. The mean body mass index (BMI) was 35.35 ± 2.16 kg/m^2^, and the mean ankle–brachial index (ABI) was 1.07 ± 0.02. The study groups were comparable in terms of age, BMI, waist circumference, and ABI values. Diabetes was the most common comorbidity, occurring in 23% of participants in each group. Renal insufficiency was present in 12% of the study group and 14% of the control group. Hypothyroidism was more than twice as prevalent in the control group compared to the study group. All patients received treatment for their comorbid conditions. Detailed population characteristics are presented in Table 2.

The study group exhibited an alpha-1 globulin concentration approximately 10% higher than that of the control group. In contrast, the mean gamma globulin level in the control group was above the reference range, whereas it remained within normal limits in the study group. Other protein fractions were similar between the two groups and fell within the established reference ranges. None of these differences reached statistical significance (Table 3).

Most mean vitamin concentrations were within the reference ranges in both groups, with occasional higher values observed in either the study or control group. The mean vitamin A level in the study group was above the reference range (1.69 ± 1.063), whereas the control group had a mean value within the normal range (0.67 ± 0.066). This difference was not statistically significant (*p* = 0.550). Vitamin D3 deficiency was observed in both groups (Table 4).

The concentrations of individual amino acids in the analyzed amino acid profile were generally similar between the two groups, with three showing statistically significant differences. Aspartic acid was elevated above the reference range in the study group, with a mean value of 5.06 ± 0.424 mg/dL compared to 3.00 ± 0.690 mg/dL in the control group (reference range < 4.0 mg/dL; *p* = 0.017). Glutamine also differed significantly, with mean values of 74.06 ± 2.580 mg/dL in the study group and 86.29 ± 6.728 mg/dL in the control group (*p* = 0.048). Methionine levels were lower in the study group (3.11 ± 0.146 mg/dL) compared to the control group (3.86 ± 0.261 mg/dL), although both values remained within the reference range (*p* = 0.015) (Table 5).

The mean values of other parameters, including free triiodothyronine (FT3), free thyroxine (FT4), hemoglobin, glycated hemoglobin (HbA1c), ferritin, homocysteine, beta-carotene, creatinine, uric acid, serum zinc, serum iron, and serum copper, were within the reference ranges for both groups. Only serum iron levels differed significantly (*p* = 0.03), with higher mean values observed in the control group (111.98 ± 15.435 µg/dL vs. 76.35 ± 7.632 µg/dL). C-reactive protein (CRP) concentrations were elevated in both groups and were more than twice as high in the study group compared with the control group (15.77 ± 4.491 mg/L vs. 6.96 ± 3.047 mg/L), although this difference did not reach statistical significance (*p* = 0.243) (Table 6).

In the study group, strong positive correlations were observed, with values above 0.8 but none exceeding 0.9. Significant positive correlations included body mass index (BMI) and waist circumference (r = 0.851, *p* < 0.05); tyrosine and proline concentrations (r = 0.840, *p* < 0.05); valine and leucine concentrations (r = 0.824, *p* < 0.05); phenylalanine and glutamic acid concentrations (r = 0.820, *p* < 0.05); and alpha-1 globulin and phenylalanine concentrations (r = 0.801, *p* < 0.05). The strongest negative correlations were observed between albumin and folic acid concentrations (r = −0.730, *p* < 0.05), albumin and vitamin B6 concentrations (r = −0.706, *p* < 0.05), folic acid and waist circumference (r = −0.650, *p* < 0.05), folic acid and BMI (r = −0.623, *p* < 0.05), and vitamin B6 and BMI (r = −0.600, *p* < 0.05). Relevant correlations in the study group are presented in Table 7.

In the control group, strong positive correlations were observed between C-reactive protein (CRP) and phenylalanine (r = 0.982, *p* < 0.05), ferritin and proline (r = 0.955, *p* < 0.05), waist circumference and proline (r = 0.955, *p* < 0.05), and tyrosine and beta-1 globulin (r = 0.954, *p* < 0.05). The most pronounced negative correlations in the control group included zinc and aspartic acid (r = −0.971, *p* < 0.05); beta-carotene and gamma globulin (r = −0.943, *p* < 0.05); total 25-hydroxyvitamin D [25(OH)D] and gamma globulin (r = −0.929, *p* < 0.05); hydroxyproline and total 25(OH)D (r = −0.926, *p* < 0.05); hydroxyproline and vitamin B1 (r = −0.926, *p* < 0.05); and hydroxyproline and vitamin B12 (r = −0.926, *p* < 0.05). Relevant correlations in the control group are summarized in Table 8.

In the study group, 24% of patients had reduced serum iron levels, with a mean value of 76.35 ± 7.632 µg/dL. Hemoglobin (Hb) levels were decreased in 18% of patients, with a mean of 13.47 ± 0.436 g/dL. No patients in the control group had reduced Hb or iron levels.

Correlation analyses between hemoglobin levels and selected parameters are presented in Table 9. In the study group, hemoglobin levels showed significant positive correlations with total protein (r = 0.61, *p* < 0.05), albumin (r = 0.65, *p* < 0.05), alanine (r = 0.49, *p* < 0.05), histidine (r = 0.61, *p* < 0.05), tryptophan (r = 0.50, *p* < 0.05), and serum iron (r = 0.76, *p* < 0.05). Significant negative correlations were observed with age (r = −0.56, *p* < 0.05) and 25(OH)D levels (r = −0.56, *p* < 0.05). In the control group, hemoglobin levels were significantly positively correlated with β1-globulins (r = 0.81, *p* < 0.05), leucine (r = 0.82, *p* < 0.05), tyrosine (r = 0.89, *p* < 0.05), valine (r = 0.78, *p* < 0.05), and serum iron (r = 0.79, *p* < 0.05). No other correlations reached statistical significance.

## 4. Discussion

The wound healing process occurs through overlapping phases, including coagulation and hemostasis, inflammation, proliferation, and remodeling. The duration of each phase is influenced by the patient’s nutritional status [7,26,31,32,47], and nutritional deficiencies can delay the healing of chronic wounds. Adequate nutrient intake is essential for effective wound repair, and the healing process itself increases the body’s demand for additional calories and protein [26,31]. Granulocytes, monocytes, and macrophages, which play key roles in wound healing, require sufficient protein to function optimally. Scientific evidence identifies four amino acids—arginine, glutamine, homocysteine, and ornithine—as particularly important for the healing process. Other factors affecting the healing of venous leg ulcers (VLUs), which have been addressed in the literature and examined in this study, are summarized in Table 1. Deficiencies in these factors may impair the healing process of venous leg ulcers (VLUs) [1,2,3,26,29,31,39,41,42]. Therefore, assessment of nutritional status should be considered an important component of the clinical evaluation of patients with chronic wounds, as correction of identified deficiencies may accelerate healing [3,26,39]. Biochemical testing may support this assessment, as selected laboratory parameters can indicate specific nutritional deficiencies. For instance, microcytic anemia may suggest iron deficiency, whereas megaloblastic anemia may indicate folate or vitamin B12 deficiency. Serum albumin is commonly used as a marker of nutritional status and should be regularly assessed and monitored in patients with chronic wounds [2].

Our study also examined comorbidities that may impair the healing of leg ulcers, including diabetes, hypothyroidism, and renal failure. Diabetes can compromise healing through tissue hypoxia, dysfunction of fibroblasts and epidermal cells, impaired neovascularization, and reduced immune function [26]. Hypothyroidism disrupts collagen synthesis by decreasing type IV collagen and hydroxyproline levels [48,49]. Renal failure affects wound healing by altering hydroxyproline levels, fibroblast proliferation, and collagen production [50,51]. Previous studies in patients with venous leg ulcers (VLUs) have assessed various proteins as indicators of nutritional status, including total protein, albumin, transferrin, C-reactive protein (CRP), and hemoglobin [1,2,3,10,11,14,26,29,31,34,36]. In the present study, we evaluated total protein, albumin, alpha-1 globulins, alpha-2 globulins, beta-1 globulins, beta-2 globulins, gamma globulins, hemoglobin, CRP, and ferritin to assess protein status. Additionally, amino acid profiles were analyzed, and correlations between different amino acids were examined in patients with VLUs. Hemoglobin, given its routine measurement and low cost, can serve as a reference marker for assessing other protein-related parameters (Table 9). To our knowledge, no previous study has simultaneously evaluated the levels of factors influencing wound healing and their interrelationships in patients with VLUs using a control group of patients with lower-limb venous insufficiency without ulcers.

The aim of increasing the number of patients in the study group was to obtain more comprehensive data on factors influencing wound healing in individuals with venous leg ulcers (VLUs). This expansion also aimed to provide additional information to identify correlations within this patient cohort. Patients with lower-limb venous insufficiency were included in the control group to evaluate differences in the levels of factors affecting wound healing between patients below clinical stage C6, according to the CEAP classification, and those with VLUs. In patients with VLUs, strong positive correlation coefficients can be used to estimate probable concentrations of selected amino acids—such as glutamic acid (r = 0.771), phenylalanine (r = 0.801), tyrosine (r = 0.762), and proline (r = 0.825)—based on alpha-1 globulin levels. Similarly, strong negative correlations allow for the prediction of vitamin B6 (r = −0.706) or folic acid (r = −0.730) concentrations from albumin levels. All these correlations were statistically significant (*p* < 0.005). In patients with lower-limb venous insufficiency without ulcers, the correlation coefficients—for example, between hydroxyproline and total 25-hydroxyvitamin D [25(OH)D] (r = −0.926), hydroxyproline and vitamin B1 (r = −0.926), and hydroxyproline and vitamin B12 (r = −0.926)—allow estimation of these vitamin concentrations from hydroxyproline levels. These correlations were also statistically significant (*p* < 0.05). The correlations presented here represent the most meaningful results obtained during the study. Given that over 3000 correlations were identified, it was not feasible to present and discuss all of them.

## 5. Study Limitations

Several limitations of the present study should be acknowledged. The relatively small sample size may have reduced the statistical power of the analyses and increased the susceptibility of the results to random variability. This limitation is particularly relevant for the correlation analyses conducted in the small control group, where correlation coefficients (r) may attain high values that do not necessarily reflect stable population-level associations and may be disproportionately influenced by individual observations. The control group included patients at different stages of the CEAP classification, which may have introduced heterogeneity into the comparative population. Given the exploratory nature of the study, which aimed to identify potential associations among the analyzed biological parameters, a large number of correlation analyses were performed. Consequently, the findings should be interpreted with caution due to the high number of statistical tests and the absence of formal correction for multiple comparisons. A further limitation of this study is the lack of detailed clinical characterization of the lesions, including their size, duration, severity, signs of infection, and the presence of non-viable tissue, which necessitates cautious interpretation of the findings. Another limitation is the absence of detailed information on treatments received in either group, including the type and regimen of therapy, adherence to compression therapy, and other wound care measures. These factors may influence clinical outcomes and laboratory parameters and thus represent potential confounders. Accordingly, the results should be interpreted with caution. Further studies involving larger and more homogeneous patient populations are required to validate the observed associations.

## 6. Conclusions

Our study identifies biochemical parallels between patients with C6 venous disease and those with non-C6 venous insufficiency. Elevated CRP levels in both groups—particularly in patients with VLUs—reflect a persistent inflammatory state, confirming CRP as a marker of inflammation. Persistently low 25(OH)D concentrations across both cohorts suggest that routine vitamin D supplementation could be considered regardless of ulcer status, although clinical discretion is warranted. By contrast, the absence of significant differences in other vitamins does not justify non-targeted supplementation without prior biochemical evaluation. Serum iron was significantly lower in the VLU group, with 24% of patients exhibiting deficiency. The strong positive correlation be-tween hemoglobin and iron levels further underscores the potential utility of routine screening and targeted iron supplementation in this population. Positive correlations between hemoglobin and iron, albumin, total protein, and selected amino acids (histidine, tryptophan) indicate that hemoglobin may serve as an indirect marker of nutritional status in patients with VLU. Negative correlations between albumin and folic acid or vitamin B6 suggest that high protein status may indicate the risk of deficiencies in these vitamins, supporting individualized supplementation strategies in VLU patients. Distinct amino acid profiles—including aspartic acid, glutamine, and methionine—together with strong correlations between biochemical parameters (e.g., albumin with B vitamins in the VLU group, hydroxyproline with vitamins D, B1, and B12 in controls) highlight the potential utility of amino acid profiling and surrogate markers in evaluating micronutrient status. Collectively, these findings provide a rationale for personalized nutritional assessment and targeted intervention in VLU patients. Further studies in larger cohorts are warranted to validate these observations and elucidate their relevance to venous leg ulcer management.

## Figures and Tables

**Table 1:** Factors affecting wound healing process and their role.

Arginine	Arginine functions as a precursor of nitric oxide, which stimulates T lymphocytes. Additionally, arginine modulates inflammatory responses, enhances immune function, facilitates angiogenesis, promotes collagen deposition, aids in wound contraction, and serves as a substrate for protein synthesis [3,26,31,32,39].
Glutamine	Glutamine plays a critical role in the inflammatory immune response during the early stages of wound healing. It serves as an essential energy source for rapidly proliferating cells, including fibroblasts, lymphocytes, macrophages, and epithelial cells [26,31,32].
Homocysteine	Elevated homocysteine levels are observed in patients with VLUs, and a reduction in these levels is associated with improved healing outcomes of the ulcers [10,16,31].
Ornithine	Ornithine promotes collagen deposition in wounds [39].
B-carotene	Beta-carotene supports antioxidant capacity, which can enhance wound healing and the formation of new tissue by leukocytes through the use of reactive oxygen species [31].
Vitamin A	Vitamin A deficiency hinders epithelialization, immune function, and collagen synthesis. Vitamin A promotes fibroblast proliferation, stimulates hyaluronate synthesis, acts as an antioxidant, and possesses anti-inflammatory properties [1,2,26,30,31,32,39].
Vitamin B1	Vitamin B1 plays a vital role as a cofactor in anabolic processes related to wound healing and in enzymatic reactions involved in leukocyte formation. It is also essential for collagen synthesis [16,32].
Vitamin B2	Vitamin B2 is a crucial cofactor involved in wound healing, anabolic processes, and enzymatic reactions related to leukocyte formation. It also plays an essential role in collagen synthesis [32].
Vitamin B6	Vitamin B6 is an important cofactor in wound healing, anabolic processes, and enzymatic reactions involved in leukocyte formation. It is also essential for collagen synthesis and may influence the healing of VLUs by reducing hyperhomocysteinemia [16,32].
Vitamin B9	Vitamin B9 is an essential cofactor in wound healing, anabolic processes, and enzymatic reactions involved in leukocyte formation. It also aids the recovery of VLUs by reducing hyperhomocysteinemia [10,32].
Vitamin B12	Vitamin B12 is a cofactor in anabolic processes involved in wound healing and in enzymatic reactions pertinent to leukocyte formation. It is crucial for collagen synthesis and may influence the healing of VLUs by reducing hyperhomocysteinemia [31,32].
Vitamin C	Vitamin C is a cofactor in the hydroxylation of lysine and proline, whose metabolites are essential for mechanical strength and collagen cross-linking. Vitamin C exhibits immunomodulatory effects and enhances the phagocytic properties of leukocytes, which can accelerate wound healing. Vitamin C deficiency can lead to reduced collagen synthesis, impaired angiogenesis, decreased fibroblast proliferation, diminished immune response, and increased susceptibility to wound infection [1,2,3,10,26,30,31,32,39,42].
Vitamin D	Vitamin D stimulates the antimicrobial peptide cathelicidin, which influences the wound healing process [31,32,43].
Vitamin E	Vitamin E accelerates the healing of venous leg ulcers, reduces inflammation, and positively affects wounds infected with methicillin-resistant *Staphylococcus aureus* (MRSA). It may also help reduce excessive scarring in chronic wounds [26,45].
Iron	Iron acts as a cofactor in the hydroxylation of proline and lysine and influences collagen synthesis. It enhances the phagocytic properties of leukocytes and supports VLU healing through its role in the oxygen transport system [2,3,26,32,39].
Zinc	Zinc is a cofactor for RNA and DNA polymerases, involved in protein and DNA synthesis. Furthermore, zinc is essential for epithelialization and collagen formation. Zinc deficiency reduces epithelialization, fibroblast proliferation, and collagen synthesis, and increases the risk of wound infection [1,3,26,31,32,39].
Copper	Copper acts as a cofactor for cytosolic antioxidant superoxide dismutase and cytochrome oxidase, contributes to proper collagen cross-linking, and enhances collagen structure [39].
Proteins	Protein deficiency delays wound healing by prolonging the inflammatory phase and inhibiting proteoglycan synthesis. The progression of the wound from the inflammatory stage to the proliferation phase is delayed due to an insufficient immune response caused by protein deficiency. Additionally, protein–calorie malnutrition reduces fibroblast activity during both the proliferation and remodeling phases, which hinders angiogenesis and the formation of new collagen [1,2,3,10,26,31,32,41].

The table presents factors described in the literature as influencing the wound healing process and briefly characterizes their impact on tissue repair.

**Table 2:** Demographic, anthropometric, and clinical characteristics of the study and control groups.

Parameter	Group 1 (Study Group) *n* = 17 Male *n* = 10 (59%) Female *n* = 7 (41%) Value	Group 2 (Control Group) *n* = 7 Male *n* = 3 (43%) Female *n* = 4 (57%) Value	*p* Value
Age (years)	63.18 ± 3.00	63.71 ± 5.639	0.806234
CEAP	C6	C1/C2/C3/C5	
ABI	1.08 ± 0.025	1.06 ± 0.042	0.589307
BMI	34.33 ± 2.596	37.83 ± 4.054	0.474211
Waist circumference (cm)	Male: 139.3 + 15.13 Female: 109.8 + 7.05	Male: 138.0 + 14.57 Female: 111.3 + 7.94	1.000000 0.915106
Hypothyroidism	*n* = 2 (12%)	*n* = 2 (29%)	
Kidney failure	*n* = 2 (12%)	*n* = 1 (14%)	
Diabetes	*n* = 5 (29%)	*n* = 2 (29%)	

Comparison of demographic, anthropometric, and clinical characteristics between patients with venous leg ulcers (study group) and patients with chronic venous insufficiency without ulcers (control group). Data are presented as mean ± standard deviation (SD) or number (percentage), as appropriate. *p*-values indicate the statistical significance of differences between the groups. Abbreviations: ABI—ankle-brachial index; BMI—body mass index; CEAP—clinical–etiology–anatomy–pathophysiology classification.

**Table 3:** Comparison of serum protein fractions between patients with venous leg ulcers and the control group.

Parameter	Reference Range	Study Group Value	Control Group Value	*p* Value
Total protein	6.40–8.20 g/dL	7.34 ± 0.126	7.57 ± 0.413	0.497
Albumin	4.02–4.76 g/dL	4.00 ± 0.108	4.03 ± 0.095	0.873
Alpha-1 globulins	0.21–0.35 g/dL	0.38 ± 0.031	0.35 ± 0.049	0.625
Alpha-2 globulins	0.51–0.85 g/dL	0.77 + 0.025	0.723 ± 0.035	0.288
Beta-1 globulins	0.34–0.52 g/dL	0.50 ± 0.013	0.51 ± 0.022	0.952
Beta-2 globulins	0.23–0.47 g/dL	0.420 ± 0.021	0.43 ± 0.031	0.696
Gamma globulins	0.8–1.35 g/dL	1.28 ± 0.076	1.53 ± 0.329	0.288

The control group consists of patients with chronic venous insufficiency without ulcers. Data are presented as mean ± standard deviation (SD). Reference ranges correspond to laboratory reference values for adult patients. *p*-values indicate the statistical significance of differences between the study and control groups.

**Table 4:** Comparison of serum vitamin levels between patients with venous leg ulcers (study group) and patients with chronic venous insufficiency without ulcers (control group).

Parameter	Reference Range	Study Group Value	Control Group Value	*p* Value
Vitamin A (retinol)	0.30–0.70 mg/L	1.69 ± 1.063	0.67 ± 0.066	0.550
Vitamin B1 (thiamine)	28.0–85.0 µg/L	59.34 ± 3.649	49.24 ± 5.187	0.140
Vitamin B2 (riboflavin)	136–370 µg/L	206.35 ± 8.282	178 ± 8.818	0.057
Vitamin B6 (pyridoxine)	5.0–30.0 µg/L	16.19 ± 2.722	11.46 ± 1.864	0.299
Vitamin B9 (folic acid)	3.1–20.5 ng/mL	7.94 ± 0.849	5.19 ± 0.755	0.065
Vitamin B12 (cyanocobalamin)	211.0–911.0 pg/mL	374.29 ± 26.72	424.57 ± 49.215	0.345
Vitamin C (ascorbic acid)	4.0–15.0 µg/mL	3.25 ± 0.587	3.94 ± 1.180	0.565
Total 25-hydroxyvitamin	30–100 ng/mL	25.23 ± 2.580	27.44 ± 3.711	0.642
Vitamin E (tocopherol)	5.0–20.0 mg/L	20.71 ± 2.040	18.77 ± 1.164	0.562

Data are presented as mean ± standard deviation (SD). Reference ranges correspond to laboratory reference values for adult patients. *p*-values indicate the statistical significance of differences between the study and control groups.

**Table 5:** Serum amino acid concentrations in patients with venous leg ulcers (study group) and patients with chronic venous insufficiency without ulcers (control group).

Parameter	Reference Range	Study Group	Control Group	*p* Value
Alanine	<51.0 mg/dL	36.65 ± 2.203	38.43 ± 2.999	0.656
Arginine	<22.0 mg/dL	16.35 ± 0.767	16.14 ± 0.594	0.869
Asparagine	<11.0 mg/dL	5.59 ± 0.243	6.00 ± 0.378	0.370
Aspartic acid	<4.0 mg/dL	5.06 ± 0.424	3.00 ± 0.690	**0.017**
Glutamine	<150.0 mg/dL	74.06 ± 2.580	86.29 ± 6.728	**0.048**
Glutamic acid	<13.0 mg/dL	19.47 ± 2.137	16.14 ± 5.369	0.490
Glycine	<32.0 mg/dL	22.53 ± 1.537	21.00 ± 2.400	0.597
Histidine	<17.0 mg/dL	9.76 ± 0.407	10.43 ± 0.571	0.376
Isoleucine	<20.0 mg/dL	8.64 ± 0.453	10.00 ± 0.787	0.133
Leucine	<25.0 mg/dL	17.82 ± 0.904	19.29 ± 1.375	0.389
Homocystin	<3.0 mg/dL	<1.0	<1.0	
Lysine	<38.0 mg/dL	23.76 ± 0.933	26.43 ± 2.08	0.188
Methionine	<6.0 mg/dL	3.11 ± 0.146	3.86 ± 0.261	**0.015**
Phenylalanine	<27.0 mg/dL	13.76 ± 1.056	13.57 ± 1.810	0.924
Serine	<17.0 mg/dL	14.35 ± 0.747	14.29 ± 1.129	0.961
Threonine	<26.0 mg/dL	11.53 ± 0.529	13.43 ± 1.130	0.096
Tyrosine	<17.0 mg/dL	10.41 ± 0.384	10.85 ± 0.986	0.610
Valine	<35.0 mg/dL	26.76 ± 1.39	28.57 ± 1.269	0.656
Beta alanine	<1.0 mg/dL	<1.0	<1.0	
Alpha aminobutyric acid	<4.0 mg/dL	1.76 ± 0.219	2.28 ± 0.421	0.242
Alpha aminoadipid acid	<2.0 mg/dL	<1.0	<1.0	
Gamma aminobutyric acid	<5.0 mg/dL	<1.0	<1.0	
Beta aminoisobutyric acid	<1.0 mg/dL	<1.0	<1.0	
Carnosine	<2.0 mg/dL	<1.0	<1.0	
Citrulline	<12.0 mg/dL	5.06 ± 0.511	5.00 ± 0.873	0.952
Homocitrulline	<2.0 mg/dL	<1.0	<1.0	
Cystathionine	<2.0 mg/dL	<1.0	<1.0	
Cystine	<34.0 mg/dL	7.82 ± 0.982	9.285 ± 1.392	0.419
Ethanolamine	<9.0 mg/dL	<1.0	<1.0	
1-methylhistidine	<7.0 mg/dL	1.00 ± 0.343	1.29 ± 9.565	0.662
3-methylhistidine	<2.0 mg/dL	0.882 ± 0.208	0.714 ± 0.184	0.634
Hydroxylysine	<10.0 mg/dL	<1.0	<1.0	
Ornithine	<30.0 mg/dL	10.82 ± 0.570	10.71 ± 0.747	0.915
Phosphoethanolamine	<6.0 mg/dL	<1.0	<1.0	
Proline	<40.0 mg/dL	21.64 ± 1.581	22.28 ± 2.532	0.831
Hydroxyproline	<7.0 mg/dL	1.94 ± 0.218	2.00 ± 0.873	0.928
Taurine	<12.0 mg/dL	15.71 ± 1.035	13.14 ± 0.911	0.153
Tryptophan	<10.0 mg/dL	9.88 ± 0.499	10.71 ± 1.392	0.484
Sarcosine	<2.0 mg/dL	<1.0	<1.0	
Phosphoserine	<2.0 mg/dL	<1.0	<1.0	

Data are presented as mean ± standard deviation (SD). Reference ranges correspond to laboratory reference values for adult patients. *p*-values indicate the statistical significance of differences between the study and control groups. Values in bold indicate statistical significance at *p* < 0.05.

**Table 6:** Comparison of additional laboratory parameters between patients with venous leg ulcers (study group) and patients with chronic venous insufficiency without ulcers (control group).

Parameter	Reference Range	Study Group	Control Group	*p* Value
Beta-carotene	150.0–1250.0 µg/L	184.43 ± 23.792	138.67 ± 17.839	0.722
Free triiodothyronine (FT3)	2.30–4.20 pg/mL	2.88 ± 0.101	2.64 ± 0.336	0.372
Free thyroxine (FT4)	0.89–1.76 ng/dL	1.08 ± 0.046	1.15 ± 0.088	0.466
Hemoglobin (Hb)	12.0–17.0 g/dL	13.47 ± 0.436	14.2 ± 0.571	0.357
Glycated hemoglobin (HbA1c)	4.50–6.20%	5.76 ± 0.133	5.82 ± 0.374	0.864
Homocysteine	5.46–16.20 µmol/L	15.07 ± 1.254	16.181 ± 3.562	0.711
CRP	<5.0 mg/L	15.77 ± 4.491	6.6 ± 3.047	0.243
Creatinine	0.4–1.3 mg/dL	0.98 ± 0.075	1.26 ± 0.426	0.351
Uric acid	3.5–7.2 mg/dL	6.451 ± 0.283	6.26 ± 0.686	0.756
Zinc	7.7–15 µmol/L	13.74 ± 0.577	15.51 ± 1.663	0.213
Copper	Male: * 18–65 years: 700–1400 μg/L * >60 years: 850–1900 μg/L Female: * 18–65 years: 800–1550 μg/L * >60 years: 850–1900 μg/L	1218.00 ± 68.33	1119.79 ± 61.382	0.399
Iron	65.0–175.0 µg/dL	76.35 ± 7.632	111.98 ± 15.435	**0.030**
Ferritin	30.0–400.0 ng/mL	120.74 ± 27.150	141.71 ± 29.77	0.658

Data are presented as mean ± standard deviation (SD). Reference ranges correspond to laboratory reference values for adult patients. *p*-values indicate the statistical significance of differences between the study and control groups. Value in bold indicates statistical significance at *p* < 0.05. Abbreviation: CRP—C-reactive protein. * Reference values stratified by age and sex.

**Table 7:** Statistically significant correlations in the study group (patients with venous leg ulcers).

Parameter	Albumin	Alpha-1 Globulins	Alpha-2 Globulins	Glutamic Acid	Leucine	Lysine	Phenylalanine	Tyrosine	Valine	Proline	Tryptophan	Vitamin C	Vitamin B6	Folic Acid
**BMI**	0.708333 *	0.260443	0.075078	0.093424	−0.007440	−0.233776	0.050682	0.418834	0.076167	0.208846	−0.050138	−0.235294	−0.600490 *	−0.622549 *
**Waist circumference**	0.689616 *	0.404806	−0.006173	0.278668	0.181599	−0.165016	0.177932	0.450221	0.122612	0.274184	0.125731	−0.393364	−0.531041 *	−0.650280 *
**Albumin**	1.000000	0.328011	−0.124309	0.263062	−0.031000	−0.269647	0.155754	0.525105 *	0.152335	0.292384	−0.120331	−0.397059	−0.705882 *	−0.730392 *
**Alpha-1 globulins**	0.328011	1.000000	−0.076496	0.77141 *	0.552541 *	0.198393	0.800511 *	0.762022 *	0.442118	0.824507 *	0.612560 *	−0.318183	0.216217	−0.082310
**Alpha-2 globulins**	−0.124309	−0.076496	1.000000	−0.090124	0.424669	0.432303	−0.041589	−0.090407	0.367675	−0.144356	−0.127145	−0.014769	−0.082462	−0.183386
**Glutamic acid**	0.263062	0.771411 *	−0.090124	1.000000	0.554747 *	0.256829	0.819603 *	0.699159 *	0.359211	0.775724 *	0.667631 *	−0.485559 *	0.041795	0.003688
**Leucine**	−0.031000	0.552541 *	0.424669	0.554747 *	1.000000	0.710890 *	0.543467 *	0.568014 *	0.824150 *	0.542596 *	0.644932 *	−0.368283	0.350923	−0.050840
**Lysine**	−0.269647	0.198393	0.432303	0.256829	0.710890 *	1.000000	0.254523	0.298440	0.659035 *	0.212652	0.363093	−0.075452	0.504659 *	0.393338
**Phenylalanine**	0.155754	0.800511 *	−0.041589	0.819603 *	0.543467 *	0.254523	1.000000	0.732077 *	0.261467	0.778205 *	0.705504 *	−0.365898	0.122378	0.069224
**Tyrosine**	0.525105 *	0.762022 *	−0.090407	0.699159 *	0.568014 *	0.298440	0.732077 *	1.000000	0.51323 *	0.839728 *	0.678390 *	−0.521354 *	−0.045009	−0.293809
**Valine**	0.152335	0.442118	0.367675	0.359211	0.824150 *	0.659035 *	0.261467	0.51323 *	1.000000	0.393473	0.409002	−0.142507	0.305898	−0.170762
**Proline**	0.292384	0.824507 *	−0.144356	0.775724 *	0.542596 *	0.212652	0.778205 *	0.839728 *	0.393473	1.000000	0.707429 *	−0.541771 *	0.208846	−0.097052
**Tryptophan**	−0.120331	0.612560 *	−0.127145	0.667631 *	0.644932 *	0.363093	0.705504 *	0.678390 *	0.409002	0.707429 *	1.000000	−0.416146	0.398598	0.115318
**Vitamin C**	−0.397059	−0.318183	−0.014769	−0.485559 *	−0.368283	−0.075452	−0.365898	−0.521354 *	−0.142507	−0.541771 *	−0.416146	1.000000	0.215686	0.394608
**Vitamin B6**	−0.705882 *	0.216217	−0.082462	0.041795	0.350923	0.504659 *	0.122378	−0.045009	0.305898	0.208846	0.398598	0.215686	1.000000	0.703431 *
**Folic acid**	−0.73039 *	−0.082310	−0.183386	0.003688	−0.050840	0.393338	0.069224	−0.293809	−0.170762	−0.097052	0.115318	0.394608	0.703431 *	1.000000

This table illustrates Spearman rank correlation coefficients (ρ) between the selected clinical and laboratory parameters in study group. Values marked with an asterisk (*) indicate the statistical significance (*p* < 0.05). Positive coefficients indicate direct correlation; negative coefficients indicate inverse correlation. Abbreviation: BMI—body mass index.

**Table 8:** Statistically significant correlations among analyzed parameters in patients with chronic venous insufficiency without ulcers (control group).

Parameter	Ferritin	Homocysteine	Alanine	Asparagine	Aspartic Acid	Leucine	Phenylalanine	Tyrosine	Proline	Hydroxyproline	Serum Zinc	Vitamin [25(OH)D]	Vitamin B1	Vitamin B12	Beta-Carotene	FT3
**BMI**	0.612637	0.43245	0.180187	−0.080403	0.626936	0.324337	0.844072 *	0.458735	0.718182	0.123443	−0.126131	−0.054056	0.000000	−0.378394	0.405840	0.054056
**Waist circumference**	0.892857 *	0.678571	0.571429	0.079682	0.617914	0.464286	0.691023	0.527360	0.954993 *	0.524631	−0.428571	−0.428571	−0.250000	−0.714286	−0.028571	−0.142857
**Beta-1 globulins**	0.558581	0.306319	0.684712	0.502519	0.176547	0.918956 *	0.614705	0.954168 *	0.445455	0.454013	0.018019	−0.684712	−0.018019	−0.414431	−0.492805	0.558581
**Gamma globulin**	0.428571	0.642857	0.785714 *	0.896421 *	0.26482	0.750000	0.509175	0.691023	0.342356	0.771517	−0.464286	−0.928571 *	−0.535714	−0.678571	−0.942857 *	0.035714
**Ferritin**	1.000000	0.571429	0.678571	0.179284	0.559065	0.678571	0.527360	0.691023	0.954993 *	0.617213	−0.428571	−0.607143	−0.178571	−0.750000	−0.142857	0.035714
**Homocysteine**	0.571429	1.000000	0.607143	0.657376	0.794461	0.321429	0.672838	0.290957	0.666694	0.925820 *	−0.857143 *	−0.678571	−0.821429 *	−0.892857 *	−0.600000	−0.607143
**CRP**	0.535714	0.607143	0.285714	0.318728	0.706188	0.464286	0.981980 *	0.563730	0.630656	0.370328	−0.285714	−0.285714	−0.250000	−0.500000	−0.028571	0.000000
**Alanine**	0.678571	0.607143	1.000000	0.557773	0.088273	0.750000	0.327327	0.709208	0.594619	0.833238 *	−0.357143	−0.928571 *	−0.392857	−0.714286	−0.828571 *	0.071429
**Asparagine**	0.179284	0.657376	0.557773	1.000000	0.254257	0.537853	0.385434	0.436149	0.180907	0.833333 *	−0.537853	−0.776899 *	−0.776899 *	−0.537853	−0.925820 *	−0.219125
**Aspartic acid**	0.559065	0.794461	0.088273	0.254257	1.000000	0.176547	0.761279	0.134343	0.597081	0.516185	−0.971008 *	−0.26482	−0.411943	−0.882735 *	−0.158114	−0.735612
**Leucine**	0.678571	0.321429	0.750000	0.537853	0.176547	1.000000	0.436436	0.981980 *	0.576600	0.617213	−0.035714	−0.750000	−0.142857	−0.428571	−0.485714	0.500000
**Phenylalanine**	0.52736	0.672838	0.327327	0.385434	0.761279	0.436436	1.000000	0.527778	0.596355	0.454013	−0.400066	−0.363696	−0.290957	−0.600099	−0.147122	−0.072739
**Tyrosine**	0.691023	0.290957	0.709208	0.436149	0.134343	0.98198 *	0.527778	1.000000	0.596355	0.508513	0.036370	−0.672838	−0.036370	−0.400066	−0.376851	0.563730
**Proline**	0.954994 *	0.666694	0.594619	0.180907	0.597081	0.576600	0.596355	0.596355	1.000000	0.626224	−0.468487	−0.504525	−0.324337	−0.720750	−0.057977	−0.126131
**Hydroxyproline**	0.617213	0.925820 *	0.833238 *	0.833333 *	0.516185	0.617213	0.454013	0.508513	0.626224	1.000000	−0.771517	−0.925820 *	−0.925820 *	−0.925820 *	−0.894427 *	−0.432049
**Tryptophan**	−0.218218	−0.763763 *	−0.218218	−0.385434	−0.787879	0.218218	−0.138889	0.305556	−0.357813	−0.688847	0.872871 *	0.309142	0.836501 *	0.618284	0.492805	0.945610 *
**Serum zinc**	−0.428571	−0.857143 *	−0.357143	−0.537853	−0.971008 *	−0.035714	−0.400066	0.036370	−0.468487	−0.771517	1.000000	0.535714	0.750000	0.857142 *	0.657143	0.785714 *
**Vitamin [25(OH)D]**	−0.607143	−0.678571	−0.928571 *	−0.776899 *	−0.26482	−0.750000	−0.363696	−0.672838	−0.504525	−0.925820 *	0.535714	1.000000	0.535714	0.785714 *	0.942857 *	0.035714

This table illustrates Spearman rank correlation coefficients (ρ) between the selected clinical and laboratory parameters in the control group. Values marked with an asterisk (*) indicate the statistical significance (*p* < 0.05). Positive coefficients indicate direct correlation; negative coefficients indicate inverse correlation. Abbreviations: BMI—body mass index; CRP—C-reactive protein; FT3—Free triiodothyronine.

**Table 9:** Correlations between hemoglobin levels and selected parameters.

Parameters	Hemoglobin: Study Group	Hemoglobin: Control Group
Age	−0.563154 *	**−0.594619**
ABI	−0.000616	**0.563730**
BMI	−0.043024	**0.576600**
Waist circumference	0.204069	**0.535714**
Total protein	0.608952 *	**0.624090**
Albumin	0.646591 *	0.214286
Alpha-1 globulins	−0.128000	0.178571
Alpha-2 globulins	−0.288971	0.178571
Beta-1 globulins	0.359606	0.810843 *
Beta-2 globulins	0.376616	0.180187
Gamma globulin	0.004920	0.357143
Glycated hemoglobin (HbA1c)	−0.340790	0.270281
Ferritin	0.444993	**0.571429**
Homocysteine	−0.282730	0.071429
CRP	−0.202828	**0.535714**
Alanine	0.489217 *	**0.535714**
Arginine	−0.129630	−0.168408
Asparagine	0.197633	0.099602
Aspartic acid	0.249749	−0.235396
Glutamine	−0.205792	**−0.607143**
Glutamic acid	0.442663	**0.750000**
Glycine	0.045090	−0.108112
Histidine	0.606938 *	**0.672838**
Isoleucine	0.038952	0.444750
Leucine	0.424767	0.821428 *
Lysine	0.185487	0.018185
Methionine	0.298348	0.079682
Phenylalanine	0.434601	0.436436
Serine	0.041538	0.444750
Threonine	0.224570	0.579066
Tyrosine	0.225738	0.891056 *
Valine	0.194701	0.781947 *
Alpha aminobutyric acid	−0.026921	**−0.752579**
Citrulline	−0.046431	0.037062
Cystine	−0.434601	**−0.673633**
Ornithine	0.132187	**0.561361**
Proline	0.408503	**0.540562**
Hydroxyproline	0.039200	0.123443
Taurine	0.148608	0.218218
Tryptophan	0.502923 *	0.472805
Creatinine	−0.212308	0.288300
Uric acid	0.468349	0.500000
Serum zinc	0.235548	0.357143
Serum copper	−0.431471	−0.142857
Serum iron	0.762142 *	0.785714 *
Vitamin A (retinol) serum	−0.333128	−0.178571
Vitamin C (ascorbic acid)	−0.199140	**−0.678571**
Total 25-hydroxyvitamin	−0.563154 *	−0.357143
Vitamin E	−0.103258	−0.714286
Vitamin B1 (thiamine)	−0.115551	0.178571
Vitamin B2 (riboflavin)	−0.356705	0.144150
Vitamin B6	0.410574	0.428571
Folic acid	0.202828	0.357143
Vitamin B12	−0.087278	−0.107143
Beta-carotene	−0.387176	0.028571
Free triiodothyronine (FT3)	0.204183	**0.678571**
Free thyroxine (FT4)	−0.401847	0.072075

This table illustrates Spearman rank correlation coefficients (ρ) between the selected clinical and laboratory parameters in both groups: patients with venous leg ulcers (study group) and patients with chronic venous insufficiency without ulcers (control group). Values marked with an asterisk (*) indicate statistical significance (*p* < 0.05). Values in bold denote strong correlations. Positive coefficients indicate direct correlation; negative coefficients indicate inverse correlation. Abbreviations: ABI—ankle-brachial index; BMI—body mass index; CRP—C-reactive protein.

## Data Availability

The datasets generated and analyzed during the current study are available from the corresponding author on reasonable request due to privacy restrictions.

## Abbreviations

The following abbreviations are used in this manuscript:

ABI	Ankle-brachial index
BMI	Body Mass Index
CEAP	Clinical–Etiology–Anatomy–Pathophysiology
CRP	C-reactive protein
VLUs	Venous leg ulcers
